# Development and Validation of a Turkish Version of Obstetric Quality of Recovery-10

**DOI:** 10.5152/TJAR.2022.21441

**Published:** 2022-10-01

**Authors:** Betül Kozanhan, Munise Yıldız, Ayşenur Polat, Oğuzhan Günenç, Sami Mahmut Tutar, Mehmet Sinan İyisoy, Nur Gözde Kulhan, Pervez Sultan

**Affiliations:** 1 Department of Anaesthesiology and Reanimation, University of Health Sciences, Konya City Hospital, Konya, Turkey; 2 Department of Obstetric and Gynecology, University of Health Sciences, Konya City Hospital, Konya, Turkey; 3 Department of Medical Education and Informatics, Necmettin Erbakan University, Konya, Turkey; 4 Stanford University Faculty of Medicine, California, USA

**Keywords:** Obstetric anaesthesia, ObsQoR-10, patient-reported outcome measure, postpartum recovery, quality of recovery

## Abstract

**Objective:**

The 10-item Obstetric Quality-of-Recovery 10 scale is a validated patient-reported outcome questionnaire that measures the quality of recovery following delivery. This study aims to develop a Turkish version of the Obstetric Quality of Recovery 10 to evaluate its validity, reliability, and clinical feasibility.

**Methods:**

Term parturients who underwent vaginal delivery or elective caesarean delivery were asked to complete a Turkish version of Obstetric Quality-of-Recovery 10 scoring tool and EuroQol 5-dimension 3L scores (including a global health visual analogue scale) 24 hours after delivery. To validate the Obstetric Quality of Recovery 10-Turkish, we assessed validity, reliability, and clinical feasibility and compared it with the EQ-5D-3L questionnaires.

**Results:**

One hundred parturients completed the questionnaire in 24 hours (100% response rate). Obstetric Quality of Recovery 10-Turkish correlated highly with EQ-5D-3L score (*r* = -0.611) and global health visual analogue scale score (*r *= 0.652) at 24 hours and discriminated well between good versus poor recovery (global health visual analogue scale score ≥70 vs <70; median interquartile range were 86 [80-90] and 68 [59-75] (*P *< .001), respectively). Scores were similar for caesarean and vaginal deliveries, 83 (76-89) and 82.5 (69-90), respectively (*P *= .5). Twenty-four-hour Obstetric Quality of Recovery 10-Turkish scores did not correlate with any baseline demographic and clinical data parameters. Internal consistency was good (Cronbach's alpha = 0.87 and inter-item correlation = 0.41), and split-half reliability was very good (Spearman-Brown prophesy reliability estimate = 0.86). Test-retest reliability was excellent (intra-class correlation coefficient = 0.99). No floor or ceiling effects were demonstrated.

**Conclusion:**

The Obstetric Quality of Recovery 10-Turkish is a valid, reliable, and clinically feasible measure of inpatient postpartum recovery following caesarean and vaginal delivery modes.

**Main Points**

- Obstetric Quality of Recovery-10 is a valid and standardised patient-reported measure.
- Obstetric Quality of Recovery 10-Turkish was evaluated in Turkish term primipara parturients following caesarean and vaginal delivery modes, and performed well in measures of validity and reliability.
- Obstetric Quality of Recovery 10-Turkish can be used in Turkish-speaking women to assess postpartum recovery.

## Introduction

Globally, 140 million women experience childbirth every year in the world.^1^ The postnatal period can impact the long-term physical, psychosocial, and emotional health of women and their infants' physical and social development since it is a critical phase in the lives of new mothers.^2^ Therefore, to improve the global quality of care and optimise the quality of recovery after delivery, defining postpartum recovery by identifying specific domains and patient-reported outcome measures (PROMs) is necessary.^3,4^

Recently, Ciechanowicz et al^5,6^ developed the "Obstetric Quality-of-Recovery (ObsQoR-11) score," an 11-item obstetric-specific questionnaire, adopted from the QoR-40, which was used to assess recovery following elective and intrapartum caesarean delivery. The ObsQoR-11 questionnaire includes evaluations of patients' physical comfort (side effects including nausea or vomiting, dizziness, shivering), pain, physical independence (ability to move independently, being able to hold and feed the baby unaided, personal hygiene/care), and emotional status (feeling in control).^5^ Obstetric Quality-of-Recovery-11 has since been modified to a 10-item version (ObsQoR-10) by Sultan et al^7-8^ and Shalev et al^9^ by combining 2 questions regarding pain into a single item based on patient feedback and validated following all modes of delivery in multiple healthcare settings.

Obstetric Quality-of-Recovery-10 has shown great potential as a PROM to provide a patient-centred, multidimensional, and standardised measure of postpartum recovery. However, ObsQoR-10 has not yet been validated in Turkish, the fifth most spoken language globally. Therefore, we intended to develop and examine the psychometric properties of a Turkish-translated version of ObsQoR-10. We hypothesised that the ObsQoR-10-Turkish would be a valid, reliable, and clinically feasible PROM for assessing postpartum recovery in a cohort of Turkish-speaking obstetric patients.

## Methods

### Patient Selection

The study protocol was approved on November 11, 2020, by the Ethics Committee of Hamidiye Scientific Researchs of University of Health Sciences Approval E-46418926-050.01.04-(20/442). The study protocol was registered at https://clinicaltrials.gov/ct2/show/NCT04729192. Written informed consent was obtained from all participating patients. In this prospective and observational study, we enrolled term obstetric patients ≥ 18 years of age and able to read and understand written Turkish at the University of Health Sciences Konya City Hospital (a university-affiliated tertiary hospital) between May and September 2021.

Inclusion criteria were primiparous patients who had singleton births with ≥38 weeks of gestational age delivering by spontaneous vaginal delivery with or without neuraxial anaesthesia for labour analgesia or via elective caesarean delivery (with spinal anaesthesia). Women were excluded if they could not read Turkish, refused to participate, or underwent an operative vaginal delivery (either vacuum or forceps delivery).

### Development of the Obstetric Quality-of-Recovery-10 Turkish Questionnaire

Permission was obtained to translate the ObsQoR-10 questionnaire^7^ to produce a Turkish version. The translation procedure was conducted in 6 steps based on the methods recommended by the EuroQoL group.^10^ In the first step, 2 independent bilingual translators, 1 experienced with health terminology, translated the English version of the ObsQoR-10 questionnaire to Turkish in a forward translation process. The differences regarding consistency and adequate vocabulary between the 2 translations were blended and compared in the second step to make the first consensus version. Then, in a back-translation process, 2 bilingual native English speakers, unfamiliar with the original version of the ObsQoR-10-Turkish, retranslated the Turkish version into English. In the fourth step, the original and back-translated versions of the questionnaire were compared by a linguist to resolve any inconsistencies. In the fifth stage, cognitive debriefing interviews were conducted with face-to-face interviews involving 10 native Turkish speakers, and ObsQoR-10-Turkish was revised based on feedback regarding question clarity and interviewee understanding. This process resulted in a translated and linguistically validated version of ObsQoR-10-Turkish (Appendix 1).

### Study Protocol

We intended to recruit a similar number of patients for caesarean and vaginal deliveries during the study period. Three investigators (OG, MY, AP) assessed the participants following delivery between 08:00 am and 05:00 pm on weekdays in the postpartum ward. The elements for enhanced recovery following caesarean delivery that our institution uses are listed in Appendix 2. Participants were invited to complete the 2 PROMs, ObsQoR-10-Turkish and EQ-5D-3L (using the validated Turkish version),^11^ 24 hours following delivery. The ObsQoR-10 questionnaire consists of 10 questions that assess the patient-reported quality of recovery in obstetric patients’ postoperative period using an 11-point Likert scale (0 = strongly negative; 10 = strongly positive) that points to a minimum score of 0 (worst possible recovery) and a maximum score of 100 (best possible recovery).^7^

The EQ-5D, one of the most widely investigated and used PROMs worldwide, consists of 5 dimensions of health: mobility, self-care, usual activities, pain/discomfort, and anxiety/depression, with 3 options selected by patients (no problem, some problems, severe problems).^12,13^ Through the interview, participants were also requested to mark their perceived global health state using a global health visual analogue scale (VAS), expressed on a 0-100 scale with 0 and 100 being the worst and best possible health states, respectively.

A subset of patients (n = 25) who were enrolled in the study answered the ObsQoR-10-Turkish and the overall postpartum recovery at 25 hours after delivery to evaluate test-retest reliability. Patients and researchers were unaware of the final survey results from the first round of survey administration at the 25-hour mark.

General characteristics of patients, including age, American Society of Anaesthesiologists Physical Status (ASA), length of hospital stay (LOS; time from delivery to discharge from hospital, measured in hours), gestation age, body mass index (BMI), mode of the delivery (spontaneous vaginal versus caesarean), duration of each stage of labour, peripartum estimated blood loss, maternal, and foetal complications were recorded at the time of enrolment for descriptive purposes.

### Data Analysis

We primarily aimed to evaluate the validity, reliability, and clinical feasibility of the ObsQoR-10-Turkish questionnaire 24 hours after delivery.

Validity as a measure of accuracy was assessed by evaluating the following:

- Convergent validity—ObsQoR-10-Turkish scores compared to the EQ-5D-3L questionnaire and 0-100 global health VAS score within 24 h after delivery.
- Discriminant validity—comparison within 24-hour ObsQoR-10-Turkish scores of women who had a “good” or “poor” postpartum recovery, defined by global VAS assessment scores of ≥70 versus <70 mm at 24 hours, respectively, and scores of women delivering via vaginal versus elective caesarean delivery.
- Hypothesis testing—correlation within 24-hour ObsQoR-10 score to the length of hospital stay (LOS; time from delivery to discharge from hospital, measured in hours), and demographic (age, BMI), obstetric factors (gestation, gestational age, duration of each stage of labour, peripartum estimated blood loss), and neonatal APGAR scores.

Reliability as a measure of consistency was assessed by evaluating the following:

- Internal consistency of ObsQoR-10-Turkish—measured using Cronbach’s alpha and inter-item correlation tests.
- Split-half reliability of final ObsQoR-10-Turkish—assessed by evaluating the correlation between random split segments.
- Test-retest reliability of ObsQoR-10-Turkish—to be assessed in 25% (assigned randomly) of women asked to repeat the questionnaire 60 min later.
- Floor and ceiling effects of ObsQoR-10-Turkish—assessed by evaluating whether <15% of respondents achieve highest (100) or lowest possible scores (0).

Acceptability and feasibility were assessed by evaluating:

- Recruitment rate of ObsQoR-10-Turkish; percentage of women agreeing to complete the study forms.
- Successful completion rate of ObsQoR-10-Turkish; the number of correctly completed ObsQoR-10-Turkish forms without missing data.
- Time taken to complete the ObsQoR-10-Turkish questionnaire; measured with a stopwatch and recorded by the investigator.

### Statistical Analysis and Sample Size

Previous ObsQoR validation studies guided the sample size for this study.^5-8^ As demonstrated previously, approximately 100 patients are needed to evaluate the psychometric properties of ObsQoR-10 and to demonstrate differences between good versus poor recovery.^5,6^ All statistical analysis was conducted using the Statistical Package for Social Sciences (SPSS) version 22.0 (IBM Corp.; Armonk, NY, USA). The normal distribution of the variables was examined with the "Kolmogorov-Smirnov test." Data are presented as mean [standard deviation (SD)], median [interquartile range (IQR)], and number (%). Correlations between ObsQoR-10-Turkish questionnaire items and global health VAS scores and correlations between scores and clinical parameters were determined using Spearman rank correlation coefficients (*r*). Internal consistency was measured with Cronbach's alpha, and split-half reliability was assessed using Spearman-Brown prophesy reliability estimate. Test-retest reliability was measured by intra-class correlation coefficient (ICC). *P* <.05 was accepted as statistically significant.

## Results

**Figure 1.**
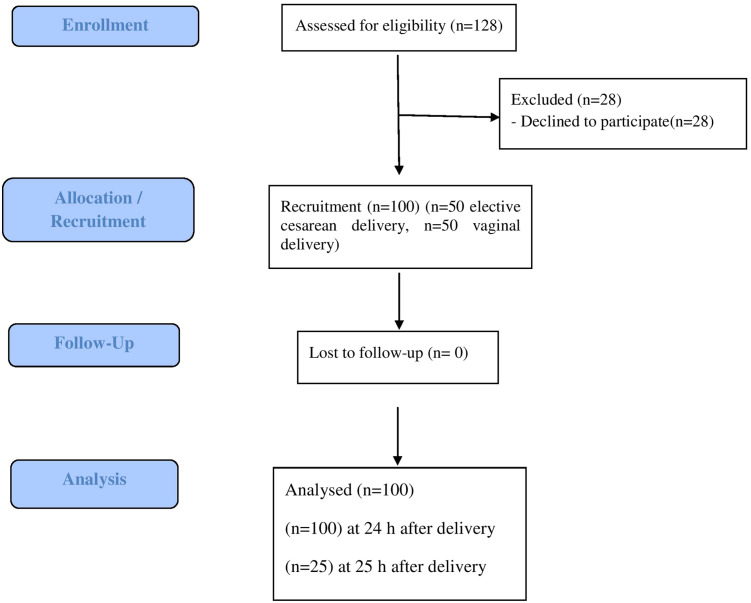
Flowchart of patient recruitment.

Between May 2021 and September 2021, 128 women were assessed for eligibility for participation in the study (Figure 1). Twenty-eight women refused to participate, and a total of 100 primiparous parturients who had singleton births completed the ObsQoR-10-Turkish 24 h after delivery.

### Demographics

The demographic and clinical characteristics of the participants are presented in Table 1. The time to complete the questionnaire was 187 (170-217.5) seconds.

### Validity

- Convergent validity: ObsQoR-10-Turkish correlated highly with EQ-5D-3L score (*r* = -0.611, *P* < .001) and global health VAS score (*r* = 0.652, *P* < .001) at 24 hours following delivery. The ObsQoR-10-Turkish and EQ-5D individual item scores are presented in Tables 2 and 3, respectively.
- Discriminant validity: The scores of ObsQoR-10 differentiated patients with good and poor postoperative recovery (VAS scores ≥70 vs. <70 at 24 hours). The median IQR ObsQoR-10-Turkish scores of patients who recovered as "good" were 86 (80-90), while those who recovered as "poor" were 68 (59-75) (*P* < .001). Scores were similar for caesarean and vaginal deliveries, 83 (76-89) and 82.5 (69-90), respectively (*P *= .593).
- Hypothesis testing: The scores of ObsQoR-10-Turkish did not correlate with LOS (*r* = -0.057, *P *= .576), demographic factors (age [*r* = -0.105, *P* = .300], BMI [*r* = 0.059, *P *= .560)], obstetric variables (gestational age [*r* = -0.158, *P *= .116], stage I duration of labour [*r* = -0.261, *P *= .068], stage II duration of labour [*r* = -0.048, *P *= .740], stage III duration of labour [*r* = -0.087, *P *= .548], peripartum estimated blood loss [*r* = -0.089, *P *= .381]), or neonatal APGAR scores (1 min *r* = 0.025, *P *= .805; 5 min *r* = .051, *P *= .615).

### Reliability

- Internal consistency of ObsQoR-10-Turkish was good, as shown by Cronbach's alpha of 0.87 for all patients and within subgroups (caesarean delivery 0.856, vaginal delivery 0.887). Interitem correlations were 0.416, as presented in Table 4.
- Split-half reliability of ObsQoR-10-Turkish was assessed with Spearman-Brown coefficient and found to be good 0.864.
- The ObsQoR-10-Turkish was repeated and completed by 25% of participants. Test-retest reliability of ObsQoR-10-Turkish was excellent with an intra-class correlation coefficient of 0.996.
- Floor and ceiling effects of ObsQoR-10-Turkish were within acceptable limits, with no participants scoring the lowest or highest values, 0 and 100, respectively.

### Clinical Feasibility

The recruitment rate for the study was 78.13% among the women screened to be eligible to participate. There was a 100% successful completion rate and no missing responses.

## Discussion

The main finding from this study is that ObsQoR-10-Turkish performs well in measures of validity, reliability, and clinical feasibility. This PROM should therefore be considered for use when evaluating the recovery of Turkish-speaking obstetric patients in the inpatient postpartum setting.

Findings from this study add further weight to the clinical validity of ObsQoR-10 as a measure of inpatient postpartum recovery. A previous U.S. study in a single-centre setting reported an ObsQoR-10 difference in score of 11 between women delivering via caesarean (elective and intrapartum) versus vaginal delivery (77 vs 86, respectively). Furthermore, the correlations demonstrated between ObsQoR-Turkish and EQ5D and global health VAS scores were stronger than that previously reported in the U.S. cohort (EQ-5D-3L *r* = 0.51 vs 0.61 and global health VAS *r* = 0.51 vs 0.65).

Childbirth is the most common indication for inpatient hospitalisation globally.^14^ The ObsQoR-10-Turkish appears to be the best currently available validated, composite, multidimensional measure of inpatient recovery for use in this population. However, despite the frequency of childbirth, postpartum recovery overall remains an underexplored area. In recent years, there has been increased interest in the importance of enhanced recovery as demonstrated by recent professional society guidelines (ERAS Society)^15-17^ and a consensus statement by the Society for Obstetric Anaesthesia and Perinatology.^18^ While postpartum recovery research has gained popularity recently, the evidence surrounding enhanced recovery protocols remains low due to heterogeneity among protocol interventions implemented and study outcomes reported.^19,20^ Consistent use of ObsQoR as a composite measure of enhanced recovery success would facilitate the pooling of results from future studies and may ultimately help strengthen the levels of evidence surrounding the use of Enhanced Recovery After Cesarean (ERAC) protocols. Furthermore, the use of ObsQoR-10 should also be considered beyond research studies, as it has been identified as the best measure of inpatient postpartum recovery, which can therefore help determine optimal peripartum management strategies within individual institutions and has clinical utility for use as an outcome measure for protocol success in quality improvement related projects.

Obstetric Quality of Recovery-10 has now been validated for use in the UK, United States, and Israeli populations, and it can be considered a representative measure for recovery across different healthcare systems and cultures.^7-9^ The development and validation of translated versions for use in lower and middle-income countries have thus far been lacking, so future studies are needed to evaluate ObsQoR-10 validity and performance in these settings.

The ObSQoR-10 has been recognised as the best currently available measure of inpatient postpartum recovery.^3,4^ A multicentre study funded by the Obstetric Anaesthesia Association within the UK recently completed recruitment of approximately 2000 women, and one aim of this study is to provide clarity regarding clinically significant values of ObsQoR-10, including scores associated with poor recovery or differences in scores corresponding to a clinically significant difference. The role of ObsQoR-10 as part of a core outcome set for use in assessing postpartum recovery still needs to be determined.

Outpatient postpartum recovery is a different construct from inpatient postpartum recovery, and the majority of a woman’s postpartum experience occurs following hospital discharge.^21^ There currently remains no robust PROM for evaluating global outpatient postpartum recovery,^22^ and the best PROMs for evaluating individual domains of postpartum recovery such as sleep and pain were not specifically developed for use in the postpartum population.^23,24^

This study was a single-centre study, and therefore, the generalisability of our findings within the wider Turkish healthcare setting requires further evaluation. However, given the consistency demonstrated thus far among the different ObsQoR-10 studies, we feel that findings from this study using ObsQoR-10-Turkish are likely to be reproducible. While ObsQoR-10-Turkish performed well in validity, reliability, and clinical feasibility measures, several psychometric properties, as described by the COSMIN group,^25^ require further study. These include measurement error, invariance, and responsiveness (evaluation of measure performance at different time points; the ability to detect change over time). Future adequately powered studies are also needed to evaluate the cross-cultural validity of ObsQoR-10. Finally, ObsQoR-10 is not designed to evaluate certain groups of women likely to experience worse recovery, such as following foetal demise, the requirement for neonatal intensive care unit (NICU) admission, maternal nerve injury, or post-dural puncture headache. Future studies should focus on recovery in these at-risk women who are more likely to experience inpatient and outpatient psychosocial morbidity.

In summary, ObsQoR-10-Turkish is a valid, reliable, and clinically feasible measure of inpatient postpartum recovery following all delivery modes that should be considered for use when evaluating the quality of recovery in Turkish-speaking obstetric patients. Future studies are needed to compare the quality of recovery between different cultures and countries using translated versions of this PROM.

## Figures and Tables

**Table 1.** Demographics of Participants

	All Participants (n = 100)	Spontaneous Vaginal Delivery (n = 50)	Caesarean Delivery (n = 50)	*P*
Age (years)	24 (22-27)	24 (21-26)	25 (22-28)	.103
BMI	28.76 (26.57-31.57)	27.79 (25.78-30.82)	29.55 (27.01-32.74)	.024*
Gestational age (year)	39 (38-40)	39 (38-40)	39 (38-40)	.663
Peripartum estimated blood loss (mL)	450 (200-750)	550 (470-550)	655 (600-800)	<.001*
Length of the hospital stay (hours)	43 (30-48)	37 (28-42)	45 (43-48)	<.001*

Results are expressed as median (25-75 percentiles) and Mann-Whitney *U* test was used. *P < .05.

**Table 2.** ObsQoR-10-Turkish Items Ranked According to Score

Ranking (Highest to Lowest)		Mean ± SD	Minimum-Maximum
1	Shivering	9.02 ± 1.33	5-10
2	Nausea or vomiting	8.48 ± 1.38	3-10
3	Feeling in control	8.34 ± 1.32	3-10
4	Dizziness	8.31 ± 1.85	3-10
5	Ability to look after personal hygiene	8.18 ± 1.87	3-10
6	Ability to feed/nurse baby without assistance	8.02 ± 2.05	2-10
7	Ability to hold baby without assistance	7.95 ± 2.02	2-10
8	Comfortable	7.85 ± 1.47	4-10
9	Ability to mobilise independently	7.76 ± 1.92	3-10
10	Pain	6.57 ± 1.90	2-10

Results are expressed as mean ± standard deviation and minimum-maximum.

**Table 3.** Summary of EQ-5D (Turkish) in Turkish Population

	EQA	EQB	EQC	EQD	EQE
1 (better recovery)	36 (36%)	34 (34%)	23 (23%)	14 (14%)	48 (48%)
2	64 (64%)	62 (62%)	74 (74%)	79 (79%)	51 (51%)
3 (worse recovery)	0 (0 %)	4 (4%)	3 (3%)	7 (7%)	1 (1%)

Results are expressed as n and percentage. EQA, movement; EQB, self care; EQC, usual activities; EQD, pain/discomfort; EQE, anxiety/depression.

**Table 4.** Inter-Class Correlation Matrix for ObsQoR-10-Turkish

Question	Global Health VAS	Total Score	QR1	QR2	QR3	QR4	QR5	QR6	QR7	QR8	QR9	QR10
QR1	0.555*	0.641*										
QR2	0.477*	0.635*	0.295*									
QR3	0.197*	0.623*	0.279*	0.368*								
QR4	0.454*	0.531*	0.400*	0.428*	0.249*							
QR5	0.671*	0.72*	0.531*	0.452*	0.310*	0.331*						
QR6	0.566*	0.797*	0.460*	0.394*	0.408*	0.298*	0.697*					
QR7	0.426*	0.771*	0.350*	0.518*	0.452*	0.344*	0.476*	0.555*				
QR8	0.461*	0.837*	0.403*	0.530*	0.543*	0.307*	0.519*	0.652*	0.868*			
QR9	0.519*	0.687*	0.438*	0.472*	0.292*	0.464*	0.453*	0.478*	0.527*	0.532*		
QR10	0.508*	0.607*	0.394*	0.450*	0.361*	0.364*	0.540*	0.525*	0.441*	0.403*	0.471*	—

Spearman correlation test. Q = item of ObsQoR-10-Turkish; VAS, visual analogue scale; ObsQoR-10, Obstetric Quality of Recovery-10.

*P < .05.

**Table:** 

*Preoperative ERAC Pathway Elements*	
1	Limit the fasting interval for solid up to before 7-8 hours and clear fluids up to 2-4 hours.
2	Patient education provided during preoperative assessment as well as antenatal care visits.
3	Education for lactation/breastfeeding provided.
4	To optimise the haemoglobin, all pregnant women are screened for anaemia per ACOG guidelines.
*Intraoperative ERAC Pathway Elements*	
1	Vasopressors and crystalloid fluid coload combination are used for the prevention and treatment of spinal-induced hypotension.
2	Preoperative patient warming, forced-air warming, and a higher ambient operating room temperature are provided to maintain normothermia.
3	To minimise side effects optimising the uterotonic dose.
4	Appropriate antibiotic prophylaxis doses are administered before skin incisions to prevent surgical site infection.
5	Using vasopressors and prophylactic intravenous antiemetics, and limiting uterine exteriorisation to prevent intraoperative and postoperative nausea and vomiting.
6	Multimodal analgesia regimen consists of applying intrathecal opioid and nonopioid analgesia (acetaminophen and nonsteroidal anti-inflammatory drug) before the onset of pain.
7	Skin-to-skin contact applied intraoperatively to the promotion of breastfeeding and maternal-infant bonding.
8	In routine cases, limit the intravenous fluids to <3 L.
9	Delay the umbilical cord clamping at least 30-60 seconds after birth to gain the specific benefits.
*Postoperative ERAC Pathway Elements*	
1	To accelerate the return of bowel function advance to regular diet within 4 hours postcaesarean, as tolerated.
2	Early ambulation after the adequate return of motor function.
3	Optimise sleep and resting periods.
4	Urinary catheter removed by 6-12 hours postpartum to facilitate ambulation, shorten hospital stay length, and lower symptomatic urinary tract infection rates.
5	Pharmacologic thromboembolism prophylaxis is used according to ACOG guidelines unless contraindicated.
6	Planning to facilitate early discharge.
7	Routinely check postpartum laboratory tests too early recognition and treatment of peripartum haemorrhage and management of postpartum anaemia.
8	Lactation education and counselling continue throughout the hospital stay to support breastfeeding.
9	Multimodal analgesia regimen consists of scheduled acetaminophen and nonsteroidal anti-inflammatory drugs. Opioids are prescribed for breakthrough pain.
10	Maintain normoglycemia.
11	To promote the return of bowel function, minimise opioid consumption, and recommend promoting mobilisation.

**Table:** 

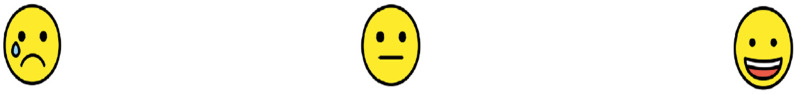		**Hayal** **edilebilecek** **en kötü Orta düzeyde Hiç yok**										
**10**	**9**	**8**	**7**	**6**	**5**	**4**	**3**	**2**	**1**	**0**
**1**	**Ağrı**											
**2**	**Bulantı veya kusma**											
**3**	Baş dönmesi											
**4**	**Titreme**											

**Table:** 

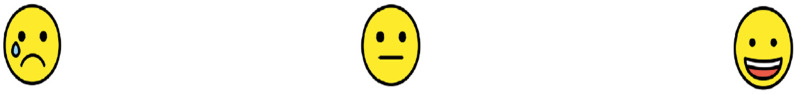		**Hayır/asla Bazen/yardımla Evet/her zaman**										
**5**	**Kendimi rahat hissediyorum**	0	1	2	3	4	5	6	7	8	9	10
**6**	**Yardım almadan hareket edebiliyorum**	0	1	2	3	4	5	6	7	8	9	10
**7**	**Yardım almadan bebeği kucağıma alabiliyorum**	0	1	2	3	4	5	6	7	8	9	10
**8**	**Bebeğimi yardım almadan besleyebiliyorum/emzirebiliyorum**	0	1	2	3	4	5	6	7	8	9	10
**9**	Kendi başıma, kişisel hijyen ve tuvalet ihtiyacımı giderebiliyorum	0	1	2	3	4	5	6	7	8	9	10
**10**	**Durumumun kontrolünün bende olduğunu hissediyorum**	0	1	2	3	4	5	6	7	8	9	10

**Table:** 

**Soru Numarası**		**ObsQoR-10 Puanı**
1	DÖNÜŞTÜRÜLMÜŞ **PUAN** **(Sayfa 1’de açıklandığı gibi 0-10 puan)**	
2		
3		
4	
5	**Sayfa 2’de girilen 0-10 puan**	
6		
7		
8		
9		
10	
**TOPLAM PUAN:**	**/100**	

## Appendix 1

**Obstetrik Derlenme Kalitesi-10 (ObsQoR-10©) Puanlama Aracı**

**Talimatlar:** Aşağıda, kadınların doğumdan sonra yaşayabileceği sorunların bir listesi bulunmaktadır. Lütfen her sorunu dikkatlice okuyun ve son 24 saat içinde bu sorundan ne kadar rahatsız olduğunuzu belirtin.

**Soru 1-4**

Lütfen son 24 saat içinde aşağıdaki semptomların şiddetini puanlayın (lütfen her semptom için bir puan (0-10) seçin).

**Araştırmacılar:** Lütfen 1-4. soruların puanlarını dönüştürün (yani 0 = 10, 1 = 9, 2 = 8, 3 = 7, 4 = 6, 5 = 5, 6 = 4, 7 = 3, 8 = 2, 9 = 1 ve 10 = 0)

**Soru 5-10**

Lütfen son 24 saatteki iyileşmenizin aşağıdaki yönlerini değerlendirin ve her madde için bir puanı daire içine alın.

**Araştırmacılar:** Lütfen toplam ObsQoR-10^©^ puanını hesaplayın.

## Appendix 2

**The Enhanced Recovery After Caesarean (ERAC) Delivery Elements Utilised in Our Institution**
